# Association between cardiovascular disease and a history of cancer in patients with chest pain on the fast track outpatient clinic

**DOI:** 10.1007/s12471-019-1268-8

**Published:** 2019-04-11

**Authors:** S. P. Sharma, M. J. Lenzen, T. W. Galema, O. C. Manintveld

**Affiliations:** Department of Cardiology, Erasmus Medical Center, University Medical Center, Rotterdam, The Netherlands

**Keywords:** Coronary artery disease, Computed tomography angiography, Cancer survivors

## Abstract

**Background:**

The purpose of this study is to investigate the prevalence of a history of malignancy in patients with chest pain who were referred for computed tomography angiography as well as the long-term survival and cardiovascular outcomes, including coronary artery disease (CAD) and coronary artery calcium (CAC) percentiles of cancer survivors. These data are relevant since it is unknown how cancer survivors, who underwent cardio-toxic therapies, should be monitored.

**Methods:**

We analysed all patients with chest pain, who came to the outpatient clinic and underwent computed tomography angiography. The primary study endpoint was long-term survival. The secondary endpoints included CAD on computed tomography angiogram (CTA), CAC percentiles, suspected and confirmed malignancy on CTA, and other accidental findings on CTA.

**Results:**

Of all 1,892 patients included in the analyses, 133 (7%) had a history of malignancy and 1,759 (93%) did not. Mortality rates were higher for the cancer survivors (6.5% vs 20.9% after ten years, *p* < 0.001). The multivariable Cox regression model also showed higher mortality for cancer survivors after ten years (adjusted hazard ratio 2.48 [95% confidence interval: 1.58–3.90]). CAD did not differ between both groups. CAC percentiles were higher in cancer survivors (*p* = 0.037). Cancer survivors had more suspected malignancies (3.8% vs 0.5%; *p* = 0.001) and also more confirmed malignancies on CTA (3.0% vs 0.1%; *p* < 0.001).

**Conclusions:**

Cancer survivors have higher mortality rates, no difference in CAD on CTA, higher CAC percentiles and more often malignancy on CTA compared with patients without a cancer history.

**Electronic supplementary material:**

The online version of this article (10.1007/s12471-019-1268-8) contains supplementary material, which is available to authorized users.

## What’s new?

- Patients with chest pain, who are referred for computed tomography angiography, with a history of malignancy show higher rates of mortality.
- Patients with a history of malignancy have no difference in coronary artery disease on computed tomography angiogram compared with patients with no history of malignancy.
- History of malignancy correlates with higher coronary artery calcium percentiles and more malignant incidental findings on computed tomography angiogram.

## Background

Cancer and cardiovascular diseases (CVD) are worldwide the two most common causes of mortality [1]. Due to improved diagnosis and treatment of cancer, the survival rate of these patients has increased over the last decades [2]. However, the treatment of cancer can cause an increase in cardiovascular morbidity. For example, a recent study suggests that coronary artery disease was significantly prevalent among Hodgkin’s lymphoma survivors, who have received cancer therapy, despite asymptomatic clinical presentation in the presence of life-threatening coronary artery disease [3]. Asymptomatic coronary artery disease can have a significant influence on the mortality and morbidity of these patients. Therefore, early diagnosis and treatment of coronary artery disease is important in cancer survivors [4]. However, guidelines on screening cancer survivors are still lacking [5, 6].

In patients with an intermediate risk of coronary artery disease, computed tomography angiography should be considered as an alternative to invasive coronary angiography in the investigation of suspected coronary artery disease [7, 8]. Computed tomography angiography is an effective, accurate, and non-invasive tool for diagnosing coronary artery disease, not only in symptomatic patients, but also in asymptomatic patients [3]. The cardiac CT scan is also used for coronary artery calcium (CAC) scoring. Just as computed tomography angiography, CAC scoring is non-invasive and can be used clinically for cardiovascular risk assessment and the investigation of patients with chest pain [9]. Moreover, computed tomography angiography and CAC scoring have the ability to depict extra cardiac lesions [10].

The primary aim of this study is to investigate whether a history of malignancy has an association with long-term survival in patients with chest pain referred for computed tomography angiography. The secondary aim is to explore the association between history of malignancy and coronary artery disease, CAC percentiles and ‘accidental’ findings (including malignancy) on computed tomography angiogram (CTA). These data are of importance since it is unknown how patients with a history of malignancy, who underwent cardio-toxic therapies, should be monitored [5, 6].

## Methods

### Study design and population

We performed a retrospective cohort study including all adult patients (>18 years) with symptoms of chest pain, who visited the outpatient clinic between September 2006 and December 2016 and who were referred for computed tomography angiography in the Erasmus MC, University Medical Centre in the Netherlands. Data on history of malignancy and the cancer treatment received were obtained from our electronic patient system. The exclusion criteria were cases that lacked data and duplicate cases. According to Dutch law, this study did not require a written informed consent.

### Study endpoints and definitions

The primary study endpoint was long-term survival. Follow-up data were obtained in 2018. We determined the patient’s survival status on 11 January 2018 by contacting the municipal civil registry. The secondary endpoints included coronary artery disease and calcium scores, malignancy on CTA and accidental findings on CTA. The coronary arteries were divided into 6 separate segments. Significant coronary artery disease was defined as coronary artery stenosis of >50%. CAC percentiles were calculated by using the CAC Score Reference Values web tool by the Multi-Ethnic Study of Atherosclerosis (MESA) to adjust for age, gender and race [11].

### Statistical analyses

Continuous variables are expressed as mean ± standard deviation (SD) values or medians [interquartile range (IQR)], depending on the distribution, and were compared by Student’s t‑test or Mann-Whitney U test. Normality was tested by the Shapiro-Wilk test. Categorical variables are expressed as frequencies and percentages and were compared by chi-squared test or Fisher’s exact test. The Kaplan-Meier method was used to evaluate the probability of survival of patients with and without a history of malignancy over 10 years. The survival curves were compared by the log-rank test. To investigate whether a history of malignancy is associated with mortality, the Cox proportional-hazards regression model was used. Variables were included in the multivariable Cox regression model, if the *p*-value was <0.20 in the univariate analysis. We have used the enter method for the Cox multiple regression model. All multivariable models were constructed using the enter method. A two-tailed *p*-value of <0.05 was considered to indicate statistical significance. All statistical analyses were performed using SPSS statistical software (IBM Corp. Released 2016. IBM SPSS Statistics for Windows, Version 24.0. Armonk, NY: IBM Corp).

## Results

### Study population

**Fig. 1:**
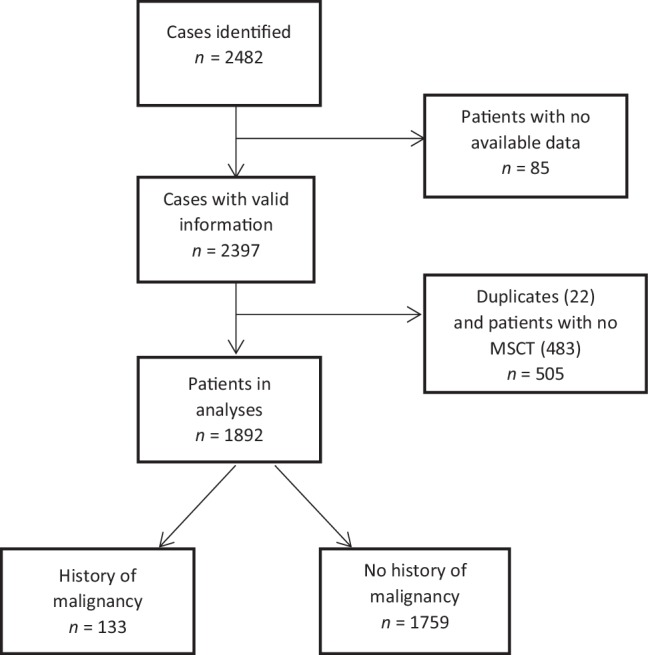
Flowchart of patients included in the study. *MSCT* multi-slice computed tomography

Of all 2,482 study cases, 85 were excluded due to lack of data, 22 cases were duplicates, and 483 patients, who had come to the outpatient clinic, were excluded because they had not undergone computed tomography angiography. Of all 1,892 patients included, 133 (7%) had a history of malignancy. (Fig. 1). Patients with a history of malignancy were significantly older (median age 61 [53–69] vs 55 [48–62] years, *p* < 0.001), were more often diagnosed with lung disease, i.e. chronic obstructive pulmonary disease (COPD) or asthma (15.8% vs 10.1%, *p* = 0.037) and were more often treated with antiplatelets (14.6% vs 9.1%, *p* = 0.034) (Tab. 1). Type of malignancy in history, cancer treatment received and median time between cancer treatment and computed tomography angiography are shown in the online Supplementary Tab. 4.

**Table 1:** Baseline characteristics

	History of malignancy *n* = 133	No history of malignancy *n* = 1,759	*P* value
Female gender, *n* (%)	71 (53.4)	913 (51.9)	0.74
Median age in years [IQR]	61.0 [53–69]	55.0 [48–62]	<0.001
Lung diseases, *n* (%)			
COPD/asthma	21 (15.8)	177 (10.1)	0.037
Cardiovascular risk factors, *n* (%)			
Hypertension	60 (45.1)	795 (45.2)	0.99
Hypercholesterolaemia	50 (37.6)	714 (40.7)	0.48
History of cardiac disease	16 (12.0)	173 (9.8)	0.42
History of invasive cardiac intervention	9 (6.8)	94 (5.3)	0.49
Creatinine >120 µmol/l	10 (5.5)	82 (3.7)	0.22
BMI >25	90 (68.7)	1,135 (67.6)	0.80
Diabetes mellitus, *n* (%)			0.70
NIDDM	16 (12)	174 (9.9)	
Insulin	7 (5.3)	108 (6.2)	
Ever smoker, *n* (%)	45 (33.8)	503 (28.6)	0.20
Medication, %			
ARB	9.5	13.6	0.17
ACE inhibitor	19.7	15.6	0.2
ASA	35	33.5	0.72
Antiplatelet drug	14.6	9.1	0.034
Beta blocker	37.2	35.6	0.70
Calcium antagonist	15.3	14.2	0.71
Diuretic	16.8	18	0.71
MRA	0	0.3	0.53
Statin/cholesterol-lowering medication	46	47.5	0.73

*COPD* chronic obstructive pulmonary disease, *BMI* body mass index, *NIDDM* non-insulin-dependent diabetes mellitus, *ARB* angiotensin II receptor blockers, *ACE inhibitor* angiotensin converting enzyme inhibitor, *ASA* acetylsalicylic acid, *MRA* mineralocorticoid receptor antagonist

### Primary outcome

**Fig. 2:**
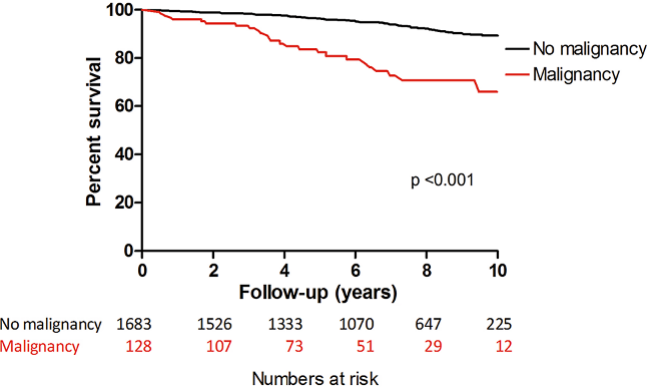
Survival curve of patients with history of malignancy versus patients with no history of malignancy in 10-year follow-up

During a median follow-up time of 7 [4.3–8.9] years, 141 (7.8%) patients died (all-cause mortality). The minimal follow-up term for patients without any event was 10 years. The survival status of 1,811 patients was known. The Kaplan Meier survival curve for all-cause mortality (Fig. 2) showed a significantly better long-term survival of patients with no history of malignancy compared with patients with a history of malignancy (all-cause mortality rate: 1.1% vs 6.2% at 2 years, 2.3% vs 12.4% at 4 years, 6.5% vs 20.9% at 10 years, overall *p* < 0.001).

Univariate analysis of the relationship with all-cause mortality identified an association with age, male gender, lung disease (COPD or asthma), history of cardiac disease, history of invasive intervention for cardiac disease, diabetes, smoking, CAC score, left ventricular function and a history of malignancy. In the multivariable analysis, factors independently associated with all-cause mortality were age, male gender, lung disease (COPD or asthma), diabetes, smoking, CAC score and a history of malignancy (Tab. 2).

**Table 2:** Univariable and multivariable predictors of all-cause mortality at 10 years follow-up

	Univariable			Multivariable		
	HR	95% CI	*P* value	HR	95% CI	*P* value
Age	1.08	1.07–1.10	<0.001	1.09	1.07–1.11	<0.001
Male gender	1.67	1.19–2.33	0.003	1.70	1.18–2.47	0.004
COPD or asthma	1.95	1.27–3.00	0.002	1.62	1.02–2.58	0.043
History of cardiac disease	2.06	1.31–3.26	0.002	1.62	0.82–3.20	0.17
History of invasive cardiac intervention	1.98	1.09–3.57	0.024	0.93	0.37–2.35	0.87
Diabetes	1.26	1.06–1.51	0.010	1.27	1.05–1.54	0.016
Smoking	1.54	1.10–2.15	0.013	1.79	1.27–1.23	0.002
LVF	2.12	1.04–4.33	0.038	1.17	0.49–2.77	0.73
CAC percentile	1.01	1.01–1.02	<0.001	1.01	1.00–1.01	0.015
History of malignancy	4.27	2.80–6.50	<0.001	2.48	1.58–3.90	<0.001

*COPD* chronic obstructive pulmonary disease, *LVF* left ventricular function, *CAC* coronary artery calcium, *HR* hazard ratio, *CI* confidence interval

### Secondary outcomes

#### Coronary artery disease on CTA and coronary artery calcium percentiles

Coronary artery disease on CTA did not differ, for any of the coronary arteries, in both groups. Patients with a history of malignancy scored higher in CAC percentiles compared with patients with no history of malignancy (52 [00–88] vs 35 [00–82], *p* = 0.037) (Tab. 3). The median absolute CAC score was also higher in patients with a history of malignancy compared with patients with no history of malignancy (18.8 [00–241.3] vs 2.7 [00–98], *p* = 0.003).

**Table 3:** CTA results

	History of malignancy *n* = 133	No history of malignancy *n* = 1,759	*P* value
CAD, *n* (%)	37 (27.8)	540 (30.7)	0.49
LM >50% stenosis, *n* (%)	1 (0.8)	13 (0.7)	1.00
LAD >50% stenosis, *n* (%)	20 (15.0)	317 (18.0)	0.39
LCX >50% stenosis, *n* (%)	10 (7.5)	83 (4.7)	0.15
RCA >50% stenosis, *n* (%)	19 (14.3)	193 (11.0)	0.24
RCX >50% stenosis, *n* (%)	7 (5.3)	41 (2.3)	0.076
Bypass graft >50% stenosis, *n* (%)	0 (0.0)	1 (0.1)	1.00
Triple-vessel disease, *n* (%)	1 (0.8)	15 (0.9)	1.00
CAC percentile^a^, median [IQR]	52 [00–88]	35 [00–82]	0.037
CAC score^b^, median [IQR]	18.8 [00–241.3]	2.7 [00–98]	0.003
Accidental findings, *n* (%)	34 (25.6)	380 (21.6)	
Aortic aneurysm, *n* (%)	0 (0.0)	30 (1.7)	0.27
Abnormal lymph nodes, *n* (%)	1 (0.8)	17 (1.0)	1.00
Diaphragmatic hernia, *n* (%)	1 (0.8)	31 (1.8)	0.72
Heart defects, *n* (%)	5 (3.8)	43 (2.4)	0.38
Liver nodule/cyst, *n* (%)	0 (0.0)	27 (1.5)	0.25
Lung nodule, *n* (%)	5 (3.8)	89 (5.1)	0.51
Skeletal abnormalities, *n* (%)	1 (0.8)	38 (2.2)	0.52
Other lung disorders, *n* (%)	5 (3.8)	60 (3.4)	0.80
Other, *n* (%)	7 (5.3)	36 (2.0)	0.016
Suspected malignancy, *n* (%)	5 (3.8)	8 (0.5)	0.001
Malignancy, *n* (%)	4 (3.0)	1 (0.1)	<0.001

*CTA* computed tomography angiography, *CAD* coronary artery disease, *LM* left main artery, *LAD* left anterior descending, *LCX* left circumflex, *RCA* right coronary artery, *RCX* right circumflex, *CAC* coronary artery calcium

^a^Out of 1,707 patients

^b^Out of 1,776 patients

#### Accidental findings on CTA

**Fig. 3:**
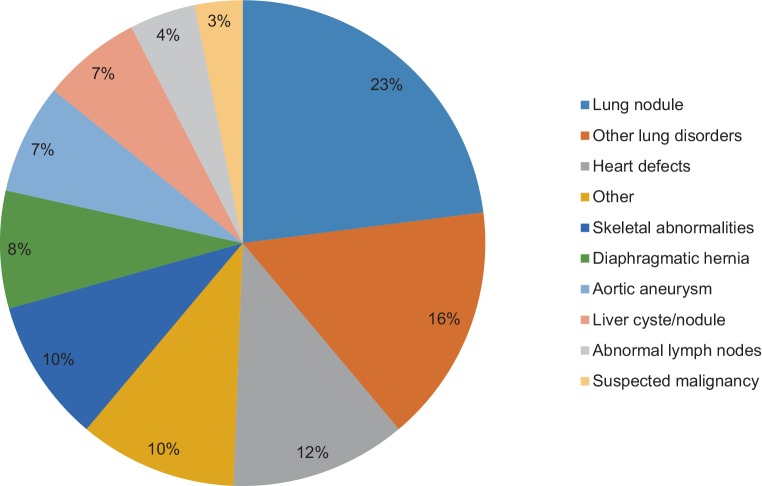
Accidental findings with computed tomography angiography

Of the patients who had undergone computed tomography angiography, 349 (18.4%) patients had an accidental finding on the CTA. As some patients had multiple accidental findings, a total of 409 (21.6%) accidental findings were found. Lung nodules were the most frequent accidental finding (94 lung nodules, 23%) (Fig. 3). The patients with a history of malignancy had a significantly higher frequency of suspected malignancy on CTA compared with patients with no history of malignancy (3.8% vs 0.5%, *p* = 0.001). After examining the pathology results, more malignancies were diagnosed in the patients with a history of malignancy (3.0% vs 0.1%, *p* < 0.001). ‘Other’ accidental findings were also more prevalent in the patients with a history of malignancy (5.3% vs 2.0%, *p* = 0.016). The rest of the categories of accidental findings on CTA were not significantly different in both groups (Tab. 3).

## Discussion

### Interpretation of findings

Current literature shows that cancer survivors are a subgroup of patients who are, due to the cancer therapy, at greater risk of developing cardiovascular disease [12]. Even though there is increasing interest in cardio-oncology, data-driven guidelines on screening cancer survivors are lacking. This is due to the small study sizes and low cardiac event rates over a short period of time [5, 6]. We performed this study to investigate the long-term outcomes of cancer survivors. In this study, we analysed the association between cardiovascular disease and a history of malignancy in a large cohort. Our study shows that outpatients with a history of malignancy who are assessed by computed tomography angiography show no difference in coronary artery disease on the CTA for any of the coronary arteries and have higher CAC percentiles and higher mortality rates compared with patients with no history of malignancy. Furthermore, accidental findings on the CTA in patients with no history of malignancy are mostly non-cancerous as opposed to patients with a history of malignancy.

Studies on childhood or adolescent cancer show that cancer survivors have higher mortality rates. However, the studies show different results on all-cause mortality. Some studies show that the overall mortality is comparable to the general population, while other studies show that excess mortality is caused by both cancer and non-cancer causes [13–15]. With the available data we were able to assess long-term survival, up to 10 years, for cancer survivors. Our study showed that cancer survivors had a significantly higher mortality rate in comparison to patients who did not have a history of malignancy. Even after adjusting, a history of malignancy remained a significant independent predictor for mortality.

Cancer survivors, who received cancer therapy, undergo various therapies that are recognised as causative agents in cardiovascular disease. Some chemotherapeutic agents have a direct effect on cardiomyocytes. This involves generation of excess reactive oxygen species, accumulation of metabolites that disrupt sarcomere structure and function, and mitochondrial dysfunction [6]. Radiation therapy causes activation of an inflammatory cascade, which affects all of the structural components of the heart, including pericardium, myocardium, heart valves, coronary arteries and capillaries, and conducting system [16]. The impact of these therapies can cause the higher risk of fatal cardiovascular events among cancer survivors compared with the general population [17].

In the univariable analysis poorer left ventricular function was a predictor for mortality. However, in the multivariable analysis poorer left ventricular function was not a significant independent predictor. The left ventricular function was good in 96% of the patients. This explains why left ventricular was not a strong predictor of mortality in this study population.

In contrast to our study, a study on CAC and cardiac radiation doses in breast cancer survivors (BCS) suggests that there is no correlation between CAC and BCS treated with radiotherapy [18]. The MESA cohort study showed that the prevalence of CAC at baseline was higher in patients diagnosed with cancer during the study compared with patients without cancer [19]. In our study cancer survivors had significantly higher CAC percentiles. One factor explaining the higher CAC percentiles is that these patients were older than the patients with no history of malignancy, however, we used the MESA web tool to adjust the CAC scores for age. Another factor clarifying the higher CAC percentiles is the radiation therapy received by 38% of the cancer survivors. CAC is an expression of atherosclerosis in the coronary arteries. Local radiation is known to initiate a cascade of events that is typical of atherosclerosis [16].

Another study on asymptomatic Hodgkin’s lymphoma survivors, who underwent radiation, claims that prevalence of coronary artery disease among Hodgkin’s lymphoma survivors is high. This study justifies screening by computed tomography angiography in asymptomatic Hodgkin’s lymphoma survivors who have had mediastinal radiotherapy. Nevertheless, this study had a very limited population size of 45 patients [3]. Our study shows that symptomatic patients show significant coronary artery disease, especially in the left anterior descending and right coronary artery. However, it does not suggest any significant difference between cancer survivors and patients with no cancer history.

There is still a discrepancy regarding the potential benefits of reanalysing CTAs for accidental findings. In our study, resembling current literature, accidental findings were prevalent in the study population, especially the lung nodules [10, 20]. Cancer survivors had significantly more malignant accidental findings. Of all the suspected malignancies in cancer survivors, 80% was confirmed by pathology. For the patients with no cancer, 12.5% of the suspected malignancies were confirmed by pathology. 3% of the cancer survivors compared with 0.1% of the patients without cancer showed a malignancy on their CTA. Therefore, accidental findings on the CTA for patients with no cancer history are mostly non-cancerous. This is in line with a study that reported 0.2% malignant findings [21].

There are several limitations to our study that should be taken into consideration while interpreting the present results. First, due to the retrospective observational study design, we cannot prove causality. Second, we only have data on all-cause mortality, but to judge cancer screening modalities it would be interesting to analyse disease-specific mortality. Nevertheless, all-cause mortality is a hard endpoint that is free from bias. Data on disease-specific mortality is often less accurate. Furthermore, we did not analyse the effects of cancer therapy. This merits further research. Strengths of our study include the large sample size, long follow-up period and the possibility to carry out extended multivariable analyses.

## Conclusion

Our study concludes that patients with a history of malignancy have higher mortality rates, no difference in coronary artery disease on the CTA for any of the coronary arteries and higher CAC percentiles compared with patients with no history of malignancy. Furthermore, our study implicates that accidental findings on the CTA for patients with no cancer history are mostly non-cancerous. However, when malignancy is found this is significantly more often in patients with a history of malignancy.

## Caption Electronic Supplementary Material

Supplementary Table 4 Type of malignancy in history
