# Supplementary material for: Association between cardiovascular disease and a history of cancer in patients with chest pain on the fast track outpatient clinic
Source: Neth Heart J. 2019 Apr 11;27(7-8):362–70. doi: 10.1007/s12471-019-1268-8 (PMC6639833; doi:10.1007/s12471-019-1268-8)
Supplement: Supplementary file 1 — Supplementary Table 4 Type of malignancy in history [file 12471_2019_1268_MOESM1_ESM.docx]

| Table 4 Malignancy in history |  |
| --- | --- |
| Type of malignancy in history, *n* (%) |  |
| Breast | 25 (18.8) |
| Gastro-intestinal | 19 (14.4) |
| Prostate | 14 (10.5) |
| Hodgkin’s lymphoma | 10 (7.5) |
| Skin | 9 (6.8) |
| Lung | 8 (6.0) |
| Leukaemia | 7 (5.3) |
| Bladder | 5 (3.8) |
| Larynx | 4 (3.0) |
| Non-Hodgkin’s lymphoma | 4 (3.0) |
| Thyroid | 4 (3.0) |
| Endometrium | 3 (2.3) |
| Renal | 3 (2.3) |
| Uterus | 3 (2.3) |
| Adrenal | 2 (1.5) |
| Cervix | 2 (1.5) |
| Ovary | 2 (1.5) |
| Pancreas | 2 (1.5) |
| Astrocytoma | 1 (0.8) |
| Gallbladder | 1 (0.8) |
| Liver | 1 (0.8) |
| Meningioma | 1 (0.8) |
| Sarcoma | 1 (0.8) |
| Schwannoma | 1 (0.8) |
| Testis | 1 (0.8) |
| Cancer treatment received, *n* |  |
| Chemotherapy | 44 |
| Radiotherapy | 51 |
| Surgery | 78 |
| Immunotherapy | 1 |
| Targeted therapy | 1 |
| Median time between cancer treatment and CTA imaging in years [IQR]* | 5 [1.3-19.5] |
| *Out of 124 patients.  *CTA* computed tomography angiography, *IQR* interquartile range |  |
